# Effects of esketamine on postoperative delirium in adult patients: a systematic review and meta-analysis of randomized controlled trials

**DOI:** 10.3389/fneur.2026.1881935

**Published:** 2026-08-20

**Authors:** Xiaohui Liu, Chaolei Liu, Yali Li, Zhenfeng Yang, Jingjing Zhang, Jiaojiao Yang, Wei He, Ya Liu, Jin Li

**Affiliations:** 1 Department of Pain, The Second Hospital of Hebei Medical University, Shijiazhuang, China; 2 Department of Anesthesiology, The Second Hospital of Hebei Medical University, Shijiazhuang, China; 3 Department of Outpatient Operating Room, The Peoples Hospital of Gaocheng, Shijiazhuang, China; 4 Department of Hematology-Oncology, The Peoples Hospital of Gaocheng, Shijiazhuang, China; 5 Department of Hepatobiliary Surgery, The Second Hospital of Hebei Medical University, Shijiazhuang, China; 6 Department of Anesthesiology and Intensive Care Unit, The Second Hospital of Hebei Medical University, Shijiazhuang, China

**Keywords:** delayed neurocognitive recovery, esketamine, grading of recommendations assessment, development and evaluation, IL-6, meta-analysis, postoperative delirium, S100β

## Abstract

**Background:**

Postoperative delirium (POD) is a common perioperative complication linked to poor prognosis. As the S-enantiomer of racemic ketamine, esketamine exhibits neuroprotective potential, yet its overall efficacy and safety for perioperative neurocognitive protection await systematic confirmation.

**Methods:**

PubMed, Embase, Cochrane Library, and Web of Science were searched from inception to November 8, 2025, for randomized controlled trials (RCTs) examining esketamine’s effect on POD in patients under elective general anesthesia. Meta-analyses were conducted via Stata/MP 18.0: risk ratios (RR) with 95% confidence intervals (CI) for dichotomous data, and mean differences (MD) or standardized mean differences (Cohen’s d) with 95% CIs for continuous outcomes. Cochrane RoB 2.0 Tool assessed bias risk in included studies. The Grading of Recommendations Assessment, Development and Evaluation (GRADE) framework was adopted to evaluate the certainty of all evidence.

**Results:**

Twenty-two RCTs involving 2,860 patients were included. Perioperative esketamine administration significantly reduced POD incidence (RR = 0.57, 95% CI: 0.43 to 0.75; *p* < 0.001), and this protective effect was consistent across subgroups (age, surgical type, dosing regimen). Esketamine also decreased delayed neurocognitive recovery (dNCR) incidence (RR = 0.60, 95% CI: 0.44 to 0.82; *p* < 0.01), improved short-term postoperative recovery quality (QoR-15 score, MD = 4.80, 95% CI: 1.06 to 8.55; *p* < 0.01), and reduced serum IL-6 (Cohen’s d = −1.47, 95% CI: −2.40 to −0.55; *p* < 0.01) and S100β (Cohen’s d = −0.85, 95% CI: −1.49 to −0.20; *p* = 0.01) levels at 1 day postoperatively. Additionally, it reduced postoperative agitation, with no obvious elevation in overall adverse event incidence. Studies with follow-up < 3 days showed no significant POD benefit, and no effect on NSE levels was observed. GRADE assessment yielded low or very low certainty for all outcomes, attributed to clinical heterogeneity, single-region study population, inconsistent outcome assessment, and limited secondary data.

**Conclusion:**

Perioperative esketamine exerts favorable neuroprotective and recovery-improving effects in Chinese surgical populations, reducing POD, dNCR, postoperative neuroinflammation, neuronal injury, and agitation with a safe adverse event profile. Restricted by geographical homogeneity, substantial heterogeneity, and low GRADE evidence certainty, further high-quality international multicenter trials are required to validate and standardize its clinical application.

**Systematic review registration:**

This systematic review has been formally registered. https://www.crd.york.ac.uk/PROSPERO/view/CRD420251184190.

## Background

Postoperative delirium (POD) is a relatively common and serious complication, especially in elderly postoperative patients (1). It is a severe and emergency encephalopathy characterized by grievous cerebral dysfunction, manifesting as fluctuations in mental status, including unstable consciousness, various degrees of inattention, and disturbed sleep–wake cycles (2). Since postoperative cognitive dysfunction (POCD) was updated to postoperative neurocognitive disorder (PND) in 2018, the definition POD has also been extended from the original 3 days postoperatively to 7 days postoperatively (3). POD has long been a research focus in anesthesiology, primarily due to its association with higher odds of mortality, postoperative complications, unplanned intensive care unit admissions, length of hospital stay, and non-home discharge (4, 5). Delirium is not only a short-term prognostic concern; with the increasing recognition that it may lead to dementia, postoperative delirium has become a public health priority (6, 7). There are numerous predisposing factors for POD, such as advanced age, polypharmacy, excessive anesthetic depth, major surgery, postoperative pain, among others (2, 8).

Esketamine or S-Ketamine is the S-enantiomer of racemic ketamine with a higher affinity for N-methyl-D-aspartate (NMDA) receptors and is approximately twice as potent as racemic ketamine for analgesia (9, 10) Additionally, compared with ketamine, esketamine has fewer psychiatric adverse reactions (including hallucinations, dissociation, and mental disorders) and better cardiovascular stability, attributed to its milder stimulatory effect on the sympathetic nervous system. Recent studies have also demonstrated its therapeutic potential for depression (11), postoperative sleep Disturbance (12), postpartum depression (13), and other conditions.

Regarding the prevention of POD, research findings on esketamine remain inconsistent—some studies have demonstrated positive effects (14, 15), while others suggest no significant benefit (16, 17). A 2025 scoping review encompassing 58 trials stands as one of the most extensive syntheses of evidence on ketamine’s cognitive impacts in surgical patients to date. Its conclusions remain ambiguous, and clarifying the role—if any—that ketamine plays in preventing PND continues to be a key ongoing research priority (18). Given that the included studies comprise both randomized controlled trials (RCTs) and retrospective cohort studies, the intervention groups involve both ketamine and enantiomeric formulations, and the study endpoints include both POD and dNCR, the heterogeneity across existing trials has precluded a definitive systematic review and meta-analysis of ketamine’s perioperative cognitive effects. Therefore, this systematic review and meta- analysis aims to explore the effects of esketamine on postoperative delirium in patients undergoing general anesthesia, based on RCTs.

## Methods

The study was registered in the International Prospective Register of Systematic Reviews (PROSPERO, CRD420251184190) and we have reported the findings in accordance with the Preferred Reporting Items for Systematic Reviews and Meta- analysis (PRISMA) (19).

### Search strategy and eligibility criteria

We systematically searched the databases of PubMed, Embase, Cochrane library and Web of science for all relevant studies from inception to Novembre 08, 2025, by two authors (XHL and CLL). We used MeSH terminology and entry words in combination, including ‘esketamine’ ‘postoperative cognitive complications’ ‘neurocognitive disorder’ and emergence delirium’. Then using the Boolean operator “AND/OR” and the search terms mesh and text to combine the results.

Articles were included if they met the following criteria: (1) randomized controlled trial involving human; (2) adult patients scheduled for elective surgery under general anesthesia; (3) the study assigned participants to an esketamine group or a control group, and documented the incidence of postoperative delirium at any observed time point. (4) complete full texts were retrievable in English or Chinese languages.

### Selection and date collection process

Two researchers (XHL and CLL) independently screened the results retrieved from the literature search. First, duplicate studies were removed. Then, the titles and abstracts of all studies were reviewed. After completing the preliminary screening, the full texts were further carefully evaluated to determine the final included literature in accordance with the inclusion criteria. Any discrepancies were discussed with a third reviewer (JL) until a consensus was reached. The screening and management of literature were performed using Endnote X9 software.

Two researchers (XHL and CLL) independently extracted information using a standardized form, capturing demographic details, surgical type, interventions (dose and route of esketamine administration), comparisons, assessment and incidence of postoperative delirium. Disagreement between the investigators were discussed with a third reviewer (JL) until consensus was reached.

### Risk bias and quality of evidence assessment

Risk of bias was assessed by two reviewers (LYL and JJZ) independently, using the Cochrane risk of bias tool (RoB 2.0 Tool) (20). This risk of bias tool presents five domains of bias. For each domain there were questions with the following possible answers: “Yes,” “Probably yes,” “Probably no,” “No” and “No information” and the risk of bias was classified as “Low risk,” “Some concerns,” or “High risk” of bias. The risk of bias was assessed for each outcome. If required, a third reviewer (JL) was consulted for decision-making.

### Outcomes assessed

The primary outcome was the incidence of POD, and the secondary outcomes included dNCR, Quality of Recovery-15 (QoR-15) scores, Mini-Mental State Examination (MMSE) scores, serum concentrations of interleukin-6 (IL-6), central nervous system specific protein *β* (S100β), and neuron-specific enolase (NSE), as well as the incidence of adverse effects (nausea and vomiting, dizziness, hallucination/nightmare, agitation, pruritus, and delayed recovery).

### Certainty of evidence assessment

We evaluated the certainty of evidence for several outcomes by using the GRADE (Grading of Recommendations, Assessment, Development and Evaluation) framework (21, 22). For each body of evidence examining the same specific intervention type, we independently assessed the overall certainty as high, moderate, low, or very low, on the basis of five domains: risk of bias, inconsistency, indirectness, imprecision, and publication bias (22, 23). Two reviewers (XHL and CLL) independently assessed the certainty of evidence, with disagreements resolved through discussion or third party adjudication. We documented the rationale for downgrading or upgrading evidence certainty for each domain.

### Statistical analysis

All statistical analyses were performed using Stata/MP 18.0 (StataCorp LLC, College Station, TX, USA) following Cochrane Handbook and PRISMA guidelines. Dichotomous outcomes were analyzed using relative risk (RR) with 95% confidence intervals (CI). Continuous outcomes were assessed via mean difference (MD) or standardized mean difference (Cohen’s d) with 95% CIs. Statistical heterogeneity across included trials was quantified using the I^2^ and H^2^ statistics. Conventionally, an I^2^ value ≤50% was interpreted as low to moderate heterogeneity, warranting the use of a fixed-effect model, whereas an I^2^ value >50% indicated substantial heterogeneity and justified adoption of a random-effect model. However, given prominent clinical heterogeneity stemming from surgical categories, esketamine dosing regimens, control group protocols, statistical model selection in the present study was not solely dictated by this arbitrary I^2^ cutoff value. For the primary pooled analyses, we followed the prespecified I^2^-based modeling rule described above. To verify the robustness of key outcomes, supplementary sensitivity analyses were further performed using two alternative random-effects estimators: the Restricted Maximum Likelihood (REML) method and the Sidik-Jonkman approach. Potential publication bias was assessed through three complementary approaches: visual symmetry evaluation of funnel plots, Egger’s linear regression test under a random-effects, and nonparametric trim-and-fill analysis with a linear estimator. A two-sided *p* < 0.05 was considered statistically significant.

## Results

### Study selection

**Figure 1:**
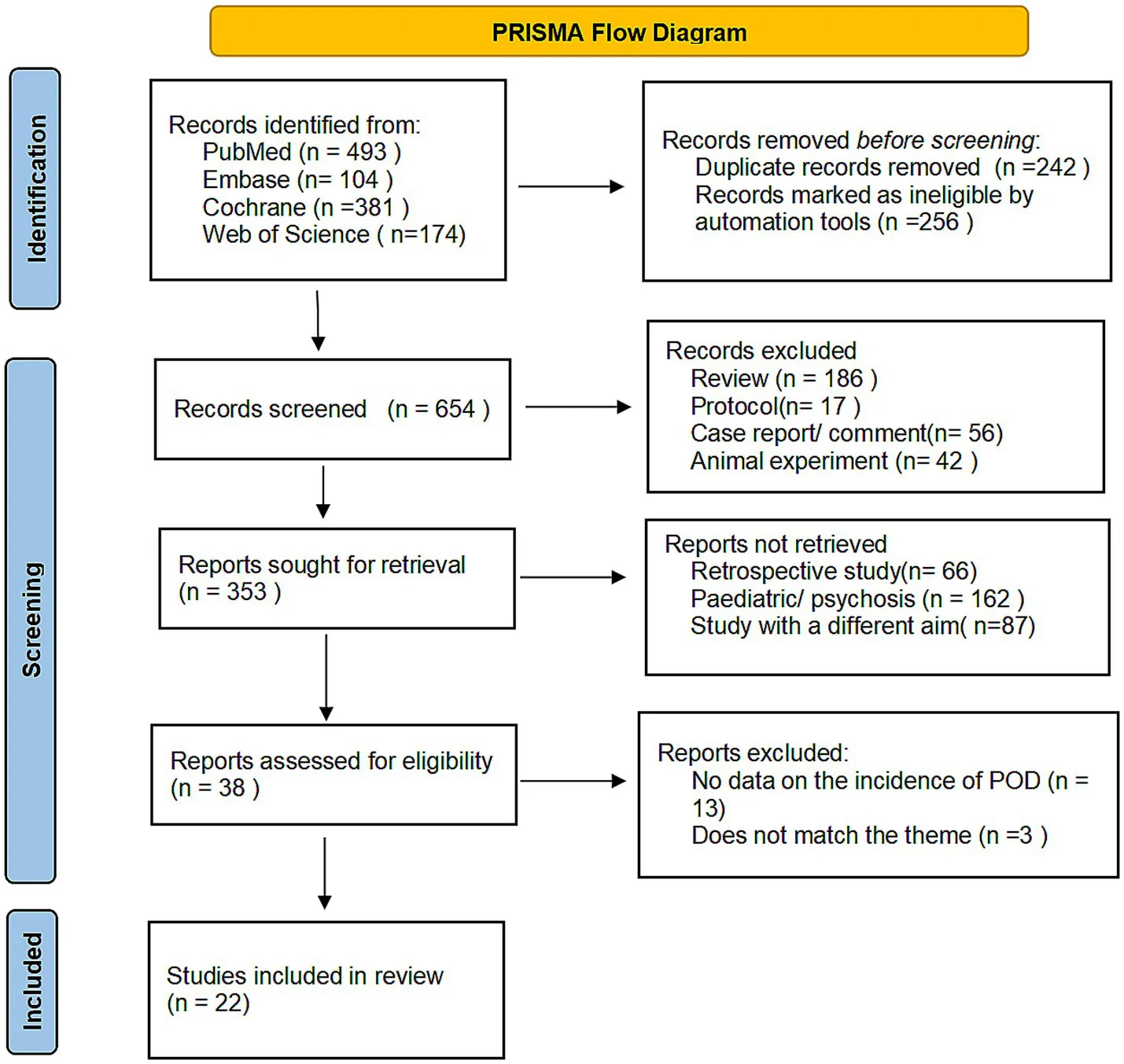
Flow chart of selection of the included studies.

Database search strategy retrieved 1,152 potentially relevant records published from 1970 to 2025. 242 duplicate studies and 256 automatically flagged trial registration records were removed. After screening the title and abstract, 616 studies did not meet the inclusion criteria. Then we excluded 16 studies after reading the full texts, including 13 studies with no data on the incidence of POD and 3 studies that did not meet our needs. Finally, there were 22 studies were left for the meta-analysis (14–17, 24–41). The flow diagram was presented in Figure 1. It follows the Preferred Reporting Items for Systematic Reviews and Meta-Analyses (PRISMA) flow diagram (19), and summarizes the reasons for exclusion of records.

### Risk of bias assessments

**Figure 2:**
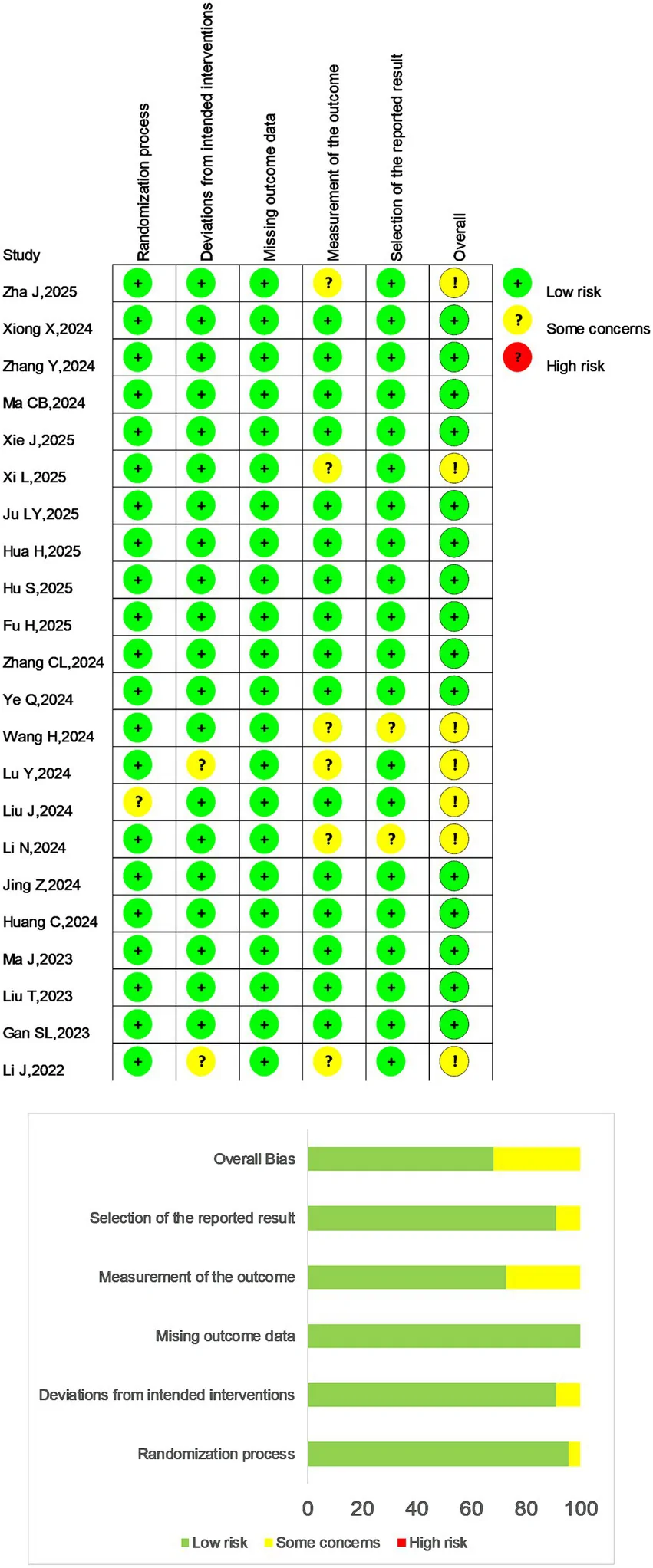
Summary of risk of bias assessment for included studies.

Risk of bias assessment revealed that 68.2% of the included studies rated as “low risk,” with no studies classified as “high risk.” The remaining 31.8% were categorized as “some concerns.” The highest risk proportion was observed in the “outcome measurement” domain, primarily attributed to study design limitations—particularly in studies where POD was a secondary outcome, with insufficient details provided regarding POD assessment methods (Figure 2).

### Characteristics of the included studies

We included 22 studies (14–17, 24–41) published between 2022 and 2025 in our analysis, encompassing a total of 2,860 patients: 1,432 in the esketamine group and 1,428 in the control group. Among these studies, 14 (63.6%) focused on elderly patients. Regarding surgical types, 3 studies involved cardiac surgery, while the remainder were major surgeries, including pulmonary surgery, gastrointestinal tumor resection, orthopedic surgery, and breast cancer surgery. The characteristics of the included studies are summarized in Table 1, such as American Society of Anesthesiologists (ASA) physical status classification, age range, surgical type, esketamine administration strategy, assessment methods and time points for POD primary and secondary endpoints, and POD incidence. In terms of dosage, most studies used subanesthetic doses of esketamine; however, its administration methods varied significantly, including single preoperative bolus injection, continuous perioperative infusion, and sole addition to patient-controlled intravenous analgesia (PCIA) pumps.

**Table 1:** Summary of the characteristics of the included studies.

Study	Surgery type	Sample size	Study group	Strategy of esketamine	Age	ASA (I/II/III/IV)	Assessment	Assessment duration (d)	Incidence of POD (%)	Endpoint priority	Effect of esketamine on POD
Zha J, 2025 (14)	Gastrointestinal tumor surgery	100	Esketamine VS normal saline	0.25 mg/kg upon anesthesia induction	Elderly	I + II/III:44/6 VS 44/6	3D-CAM	7	24.0 VS 48.0	primary	positive
Xiong X, 2024 (15)	Heart valve replacement	112	Esketamine VS normal saline	Single dose of 0.25 mg/kg before anesthesia induction	Adult	0/14/42/0 VS 0/13/43/0	CAM-ICU/3D-CAM	7	23.2 VS 42.9	primary	positive
Zhang Y, 2024 (16)	Noncardiac surgery	426	Esketamine VS normal saline	Single dose 0.2 mg/kg before anesthesia induction	Elderly	/	3D-CAM	7	54.9 VS 53.1	primary	No effect
Ma CB, 2024 (17)	TKA and THA	260	Esketamine VS normal saline	0.20 mg/kg loading, 0.125 mg/kg/h infusion, 0.5 mg/kg for postoperative analgesia	Elderly	0/96/34/0 VS 0/101/29/0	3D-CAM	3	8.5 VS 10.8	primary	no effect
Xie J, 2025 (24)	Esophageal cancer surgery	202	Esketamine VS normal saline	Continue intravenous esketamine 0.015 mg/kg/h for 48 h postoperatively.	Adult	22/77/0/0 VS 27/76/0/0	ICDSC	/	16.2 VS 13.6	Secondary	No effect
Xi L, 2025 (25)	Heart valve replacement	78	Esketamine+remimazolam VS dexmedetomidine	0.5 mg/kg before operation and maintained at 0.5 mg/kg/h intraoperatively	Adult	0/14/25/0 VS 0/11/28/0	CAM	3	12.82 VS 33.3	Secondary	positive
Ju LY, 2025 (26)	OPCABG	134	Esketamine VS normal saline	0.25 mg/kg/h intraoperatively	Adult	0/0/12/55 VS 0/0/16/51	CAM-ICU / 3D-CAM	7	13.4 VS 28.4	primary	No effect
Hua H, 2025 (27)	THA	114	Esketamine+dexmedetomidine opioids free anesthesia VS balanced anesthesia with opioids	0.35 mg/kg at induction and 0.2 mg/kg/h at maintenance	Elderly	0/35/22/0 VS 0/42/15/0	CAM-CR	3	5.0 VS 17.0	primary	positive
Hu S, 2025 (28)	Lumbar fusion surgery	78	Esketamine VS normal saline	Single dose of 0.25 mg/kg after anesthesia induction	Elderly	0/32/7/0 VS 0/30/9/0	CAM	3	15.0 VS 44.0	Secondary	positive
Fu H, 2025 (29)	Lung cancer surgery	106	Esketamine VS normal saline	0.25 mg/kg during induction and 0.1 mg/kg/h during maintenance for 30 min	Elderly	0/22/31/0 VS 0/26/27/0	CAM	3	3.8 VS 15.1	primary	positive
Zhang CL, 2024 (30)	Lung cancer surgery	163	Esketamine+dexmedetomidine VS dexmedetomidine	0.25 mg/kg esketamine during anesthesia induction,continuous infusion 0.125 mg/kg/h until 30 min before the end of surgery	Elderly	0/23/59/0 VS 0/19/62/0	3D-CAM	7	14.6 vs. 30.9	primary	positive
Ye Q, 2024 (31)	Hip surgery	121	Esketamine+dexme-detomidine opioids free group VS balanced anesthesia with opioids	Esketamine 0.5 mg/kg during induction and 0.25 mg/kg/h during maintenance	Elderly	0/44/16/0 VS 0/37/24/0	CAM	2	11.7 VS 4.8	Secondary	No effect
Wang H, 2024 (32)	Breast cancer surgery	64	Esketamine VS normal saline	single dose of 0.2 mg/kg after anesthesia induction	Adult	/	/	1	0 VS 0	Secondary	No effect
Lu Y, 2024 (33)	Thoracic surgery	94	Esketamine VS dexmedetomidine	0.5 mg/kg 20 min after anesthesia induction	Elderly	14/19/14/0 VS 12/21/14/0	CAM	/	4.3 VS 19.2	Secondary	positive
Liu J, 2024 (34)	Gastrointestinal surgery	60	Esketamine+sufenta-nil VS sufentanil in PCIA	1 mg/kg in PCIA pump	elderly	/	CAM	3	13. VS 40.0	Secondary	positive
Li N, 2024 (35)	Breast cancer surgery	120	Esketamine VS sufentanil	0.30 mg/kg loading,0.25 mg/kg/h infusion,100 mg for postoperative analgesia	Adult	41/19/0/0 VS 40/20/0/0	/	2	5.0 VS 6.7	Secondary	no effect
Jing Z, 2024 (36)	Gastrointestinal tumor surgery	87	Esketamine VS normal saline	Single dose of 0.25 mg/kg and 0.1 mg/kg/h infusion	Elderly	0/35/9/0 VS 0/34/9/0	CAM	3	2.3 VS 11.6	Secondary	No effect
Huang C,2024 (37)	Orthopedic, urologic and major abdominal surgeries	209	Esketamine VS normal saline	0.5 mg/kg 10 min after induction and 2 mg/kg for PCIA	Elderly	12/174/27 VS 8/166/30	CAM	5	12.1 VS 10.9	primary	No effect
Ma J, 2023 (38)	Gastrointestinal tumors surgery	62	Esketamine VS normal saline	0.25 mg/kg loading and 0.125 mg/kg/h infusions	elderly	0/25/6/0 VS 0/24/7/0	CAM-ICU	3	9.7 VS 12.9	Secondary	No effect
Liu T, 2023 (39)	Gynecological surgery	39	Esketamine VS normal saline	0.125 mg/kg 30 min intraoperatively	Adult	3/17/0/0 VS 1/18/0/0	CAM-ICU	1	0 VS 0	Secondary	No effect
Gan SL, 2023 (40)	Lung cancer surgery	151	Esketamine VS normal saline	0.1 mg/kg loading, 0.1 mg/kg/h infusion, 1 mg/ kg in PCIA	Adult	35/42/1 VS 32/45/1	CAM/CAM-ICU	/	0 VS 0	Secondary	No effect
Li J, 2022 (41)	TKA	80	Esketamine VS normal saline	0.2 mg/kg during induction	elderly	0/36/4/0 VS 0/35/5/0	CAM	3	0 VS 2.5	Secondary	No effect

TKA, Total knee arthroplasty; THA, Total hip arthroplasty; OPCABG, Off-pump coronary artery bypass grafting; PCIA, Patient controlled intravenous analgesia; ICDSC, Intensive Care Delirium Screening Checklist; CAM-CR, Chinese Revised Delirium Diagnostic Scale; 3D-CAM, 3-min Diagnostic Confusion Assessment Method; CAM, Confusion Assessment Method; CAM-ICU, Confusion Assessment Method for the Intensive Care Unit.

### Effect of esketamine on POD

**Figure 3:**
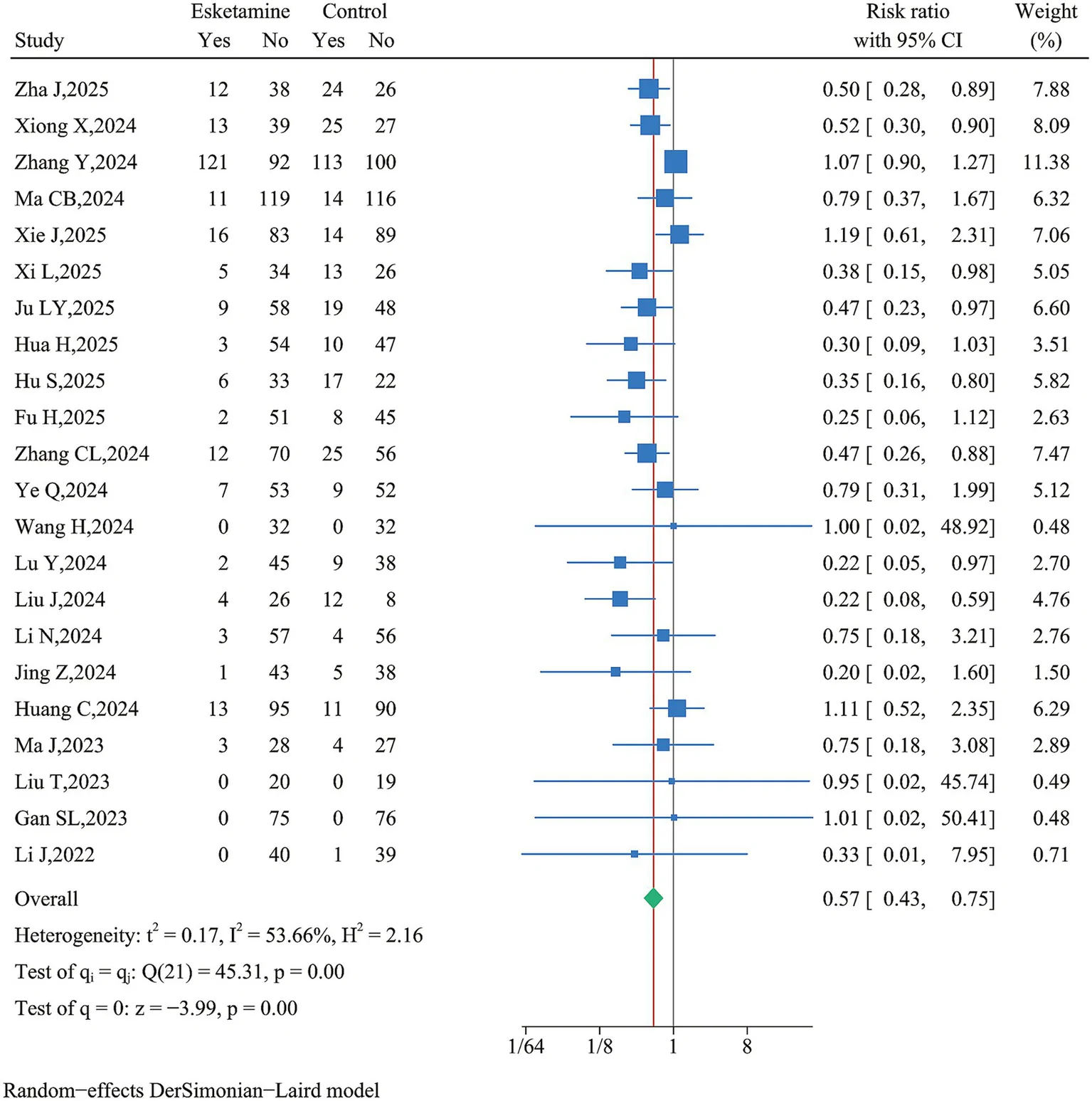
Forest plot of the incidence of POD between Esketamine and control group.

Statistical heterogeneity was detected across the 22 included studies (I^2^ = 53.66%, H^2^ = 2.16), prompting the adoption of the DerSimonian-Laird random-effects model for all meta-analyses. The pooled results demonstrated that esketamine administration was significantly associated with a reduced incidence of POD compared with the control group (RR = 0.57, 95%CI: 0.43 to 0.75; *p* < 0.001; Figure 3). Correspondingly, intraoperative esketamine use conferred a protective effect against POD, reducing the relative risk of developing POD by 43%.

### Sensitivity analyses of primary outcome

Owing to double-zero events and sparse event counts observed in several included trials, we conducted multiple sensitivity analyses to validate the robustness of our primary pooled estimates, employing the REML random-effects model and the Sidik–Jonkman variance estimator. The REML model generated a pooled RR of 0.58 (95% CI: 0.45 to 0.75; *p* < 0.001; Supplementary Figure 1), while the Sidik–Jonkman approach yielded highly consistent results, with a pooled RR of 0.57 (95% CI: 0.44 to 0.75; *p* < 0.001; Supplementary Figure 2).

For further verification, we manually excluded four trials (32, 39–41) with double-zero events and re-analyzed the remaining data using the DerSimonian–Laird random-effects model. The resulting pooled RR was 0.56 (95% CI:0.42 to 0.75; *p* < 0.001; Supplementary Figure 3), which still indicated a significant protective effect of esketamine against postoperative delirium. In addition, three trials (25, 27, 31) applied esketamine combined with remimazolam or dexmedetomidine; concomitant use of other sedatives may magnify the efficacy signal of esketamine alone. We thus removed these three combination-treatment studies to explore the pure effect of esketamine monotherapy versus placebo or control drugs. The updated pooled RR was 0.59 (95% CI:0.43 to 0.79; *p* < 0.001; Supplementary Figure 4).

The conclusions derived from sensitivity analyses were largely aligned with the main pooled results, offering suggestive evidence that our core findings were relatively stable.

### Subgroup analyses of primary outcome

**Figure 4:**
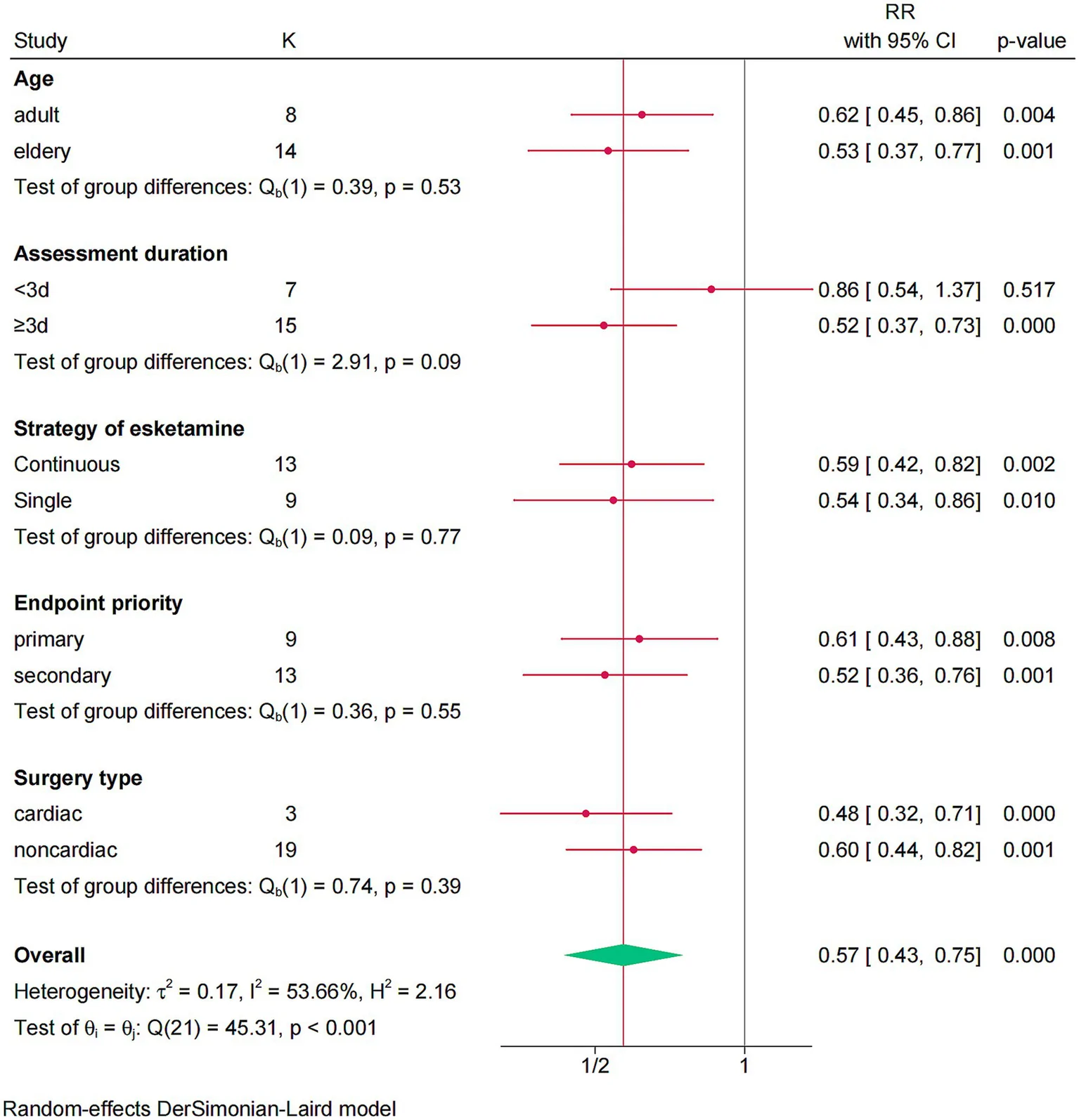
Summary of subgroup analyses for POD incidence.

To explore the sources of heterogeneity and verify the consistency of esketamine’s efficacy across varying administration routes, patient populations, and surgical types, prespecified subgroup analyses were conducted (summary presented in Figure 4). Among all subgroups, only the stratum evaluating studies with a follow-up duration of < 3 days yielded results inconsistent with the overall findings, showing no significant association between esketamine and POD risk (RR = 0.86, 95% CI: 0.54 to 1.37; *p* = 0.517, Figure 4; Supplementary Figure 5).

In contrast, all other subgroups—including those stratified by patient age (Supplementary Figure 6), esketamine administration regimen (Supplementary Figure 7), endpoint priority (Supplementary Figure 8) —yielded results consistent with the primary analysis.

**Figure 5:**
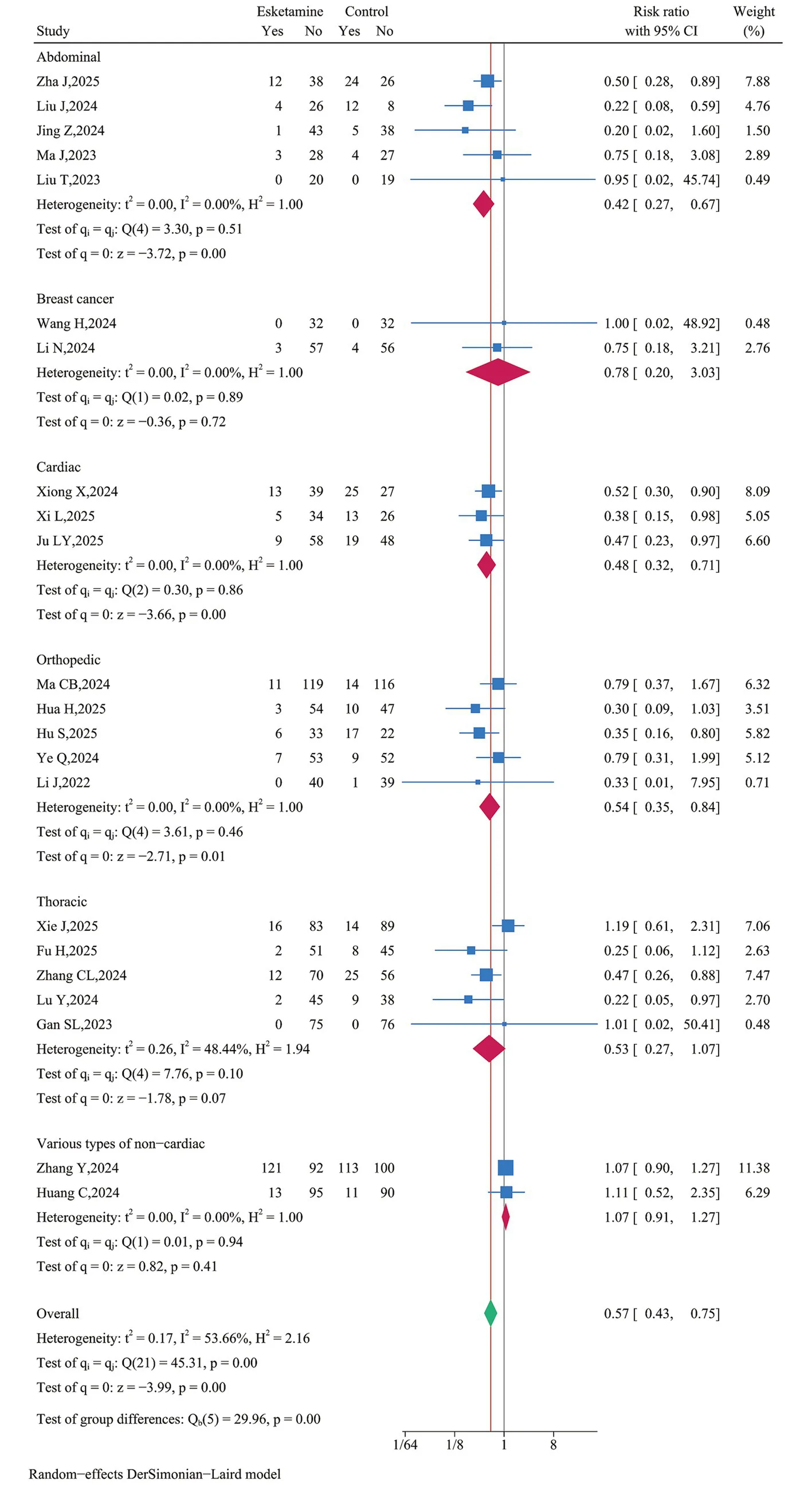
Subgroup analysis by surgical type.

We further subdivided the included studies into refined surgical subgroups to explore potential effect modification (Figure 5; Supplementary Figure 9). Subgroup analyses revealed that the magnitude of the protective effect of esketamine against postoperative delirium varied markedly across these finely categorized surgical types, indicating inconsistent treatment effects among subgroups.

### Other outcomes of interest

In several of the included articles, additional outcomes were reported, including dNCR, QoR-15 scores, MMSE scores, serum concentrations of inflammatory factors such as IL-6 and brain injury biomarkers (S100β and NSE).

**Figure 6:**
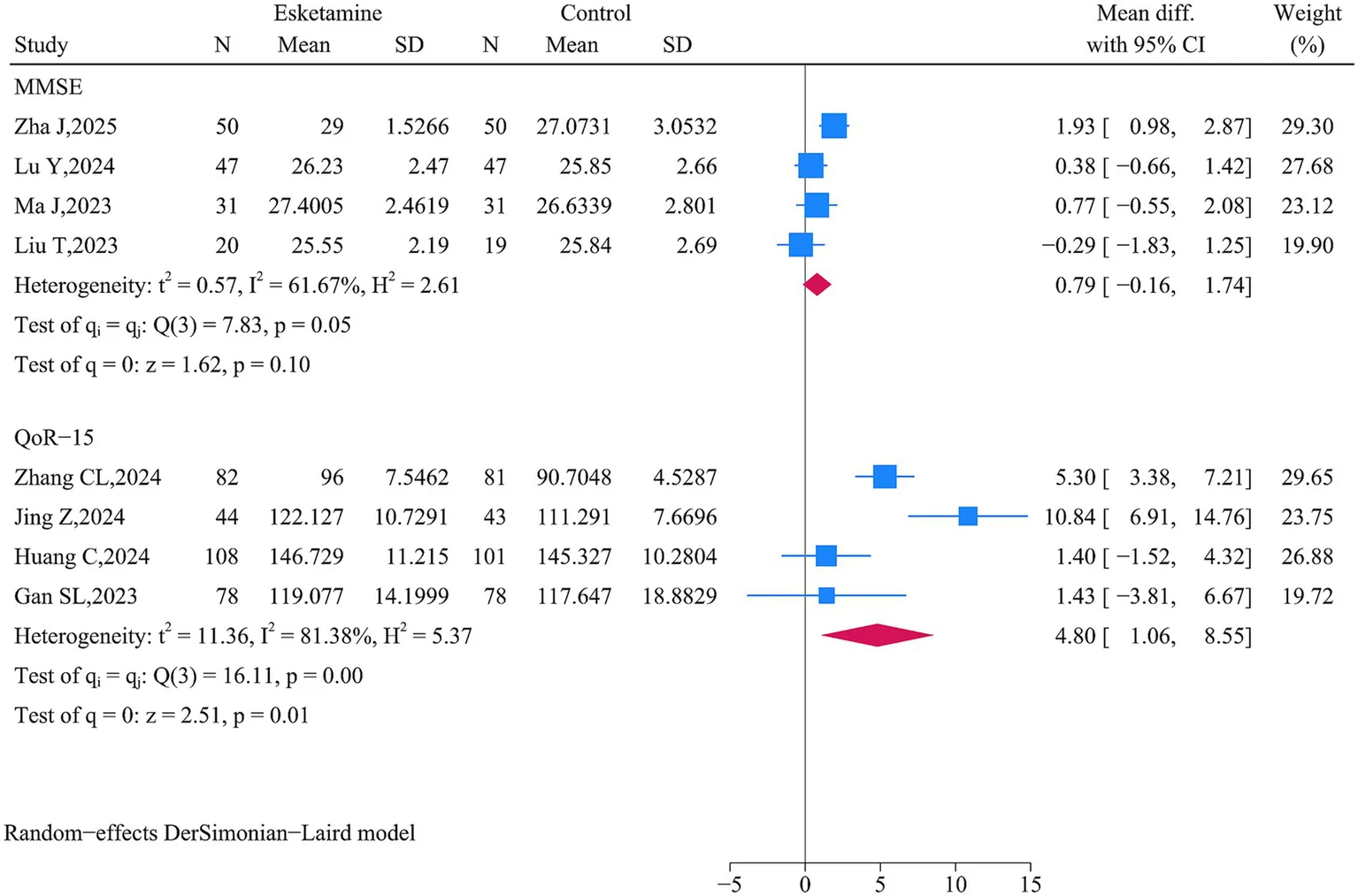
Differences in MMSE and QoR-15 scores between esketamine and control groups.

**Figure 7:**
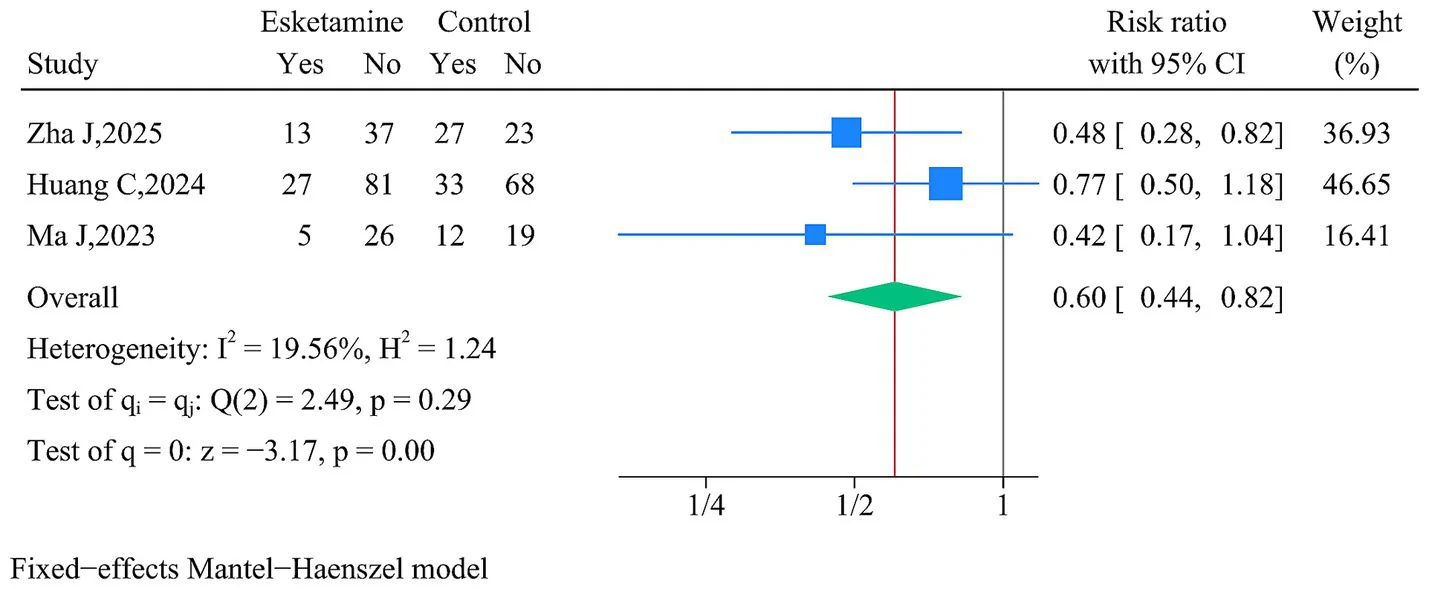
Forest plot of the incidence of dNCR between esketamine and control group.

Seven studies (14–16, 25, 33, 38, 39) further conducted postoperative MMSE assessments; however, specific score data were retrievable from only four of these studies (Figure 6). Regrettably, perioperative esketamine administration did not significantly improve MMSE scores at 1 day post-general anesthesia surgery (MD = 0.79, 95%CI: −0.16 to 1.74; *p* = 0.05). Although the improvement in absolute MMSE values lacked statistical significance, a pooled analysis of three studies (14, 37, 38) demonstrated a beneficial effect of esketamine on dNCR (RR = 0.60, 95% CI: 0.44 to 0.82; *p* < 0.01; Figure 7).

Concurrently, four studies (30, 36, 37, 40) evaluated patients’ postoperative recovery quality using the QoR-15 as the assessment tool, with follow-up assessments extending up to 90 days postoperatively. Meta-analysis revealed that intraoperative esketamine administration could enhance patients’ short-term postoperative recovery quality (MD = 4.80, 95% CI: 1.06 to 8.55; *p* < 0.01; Figure 6).

**Figure 8:**
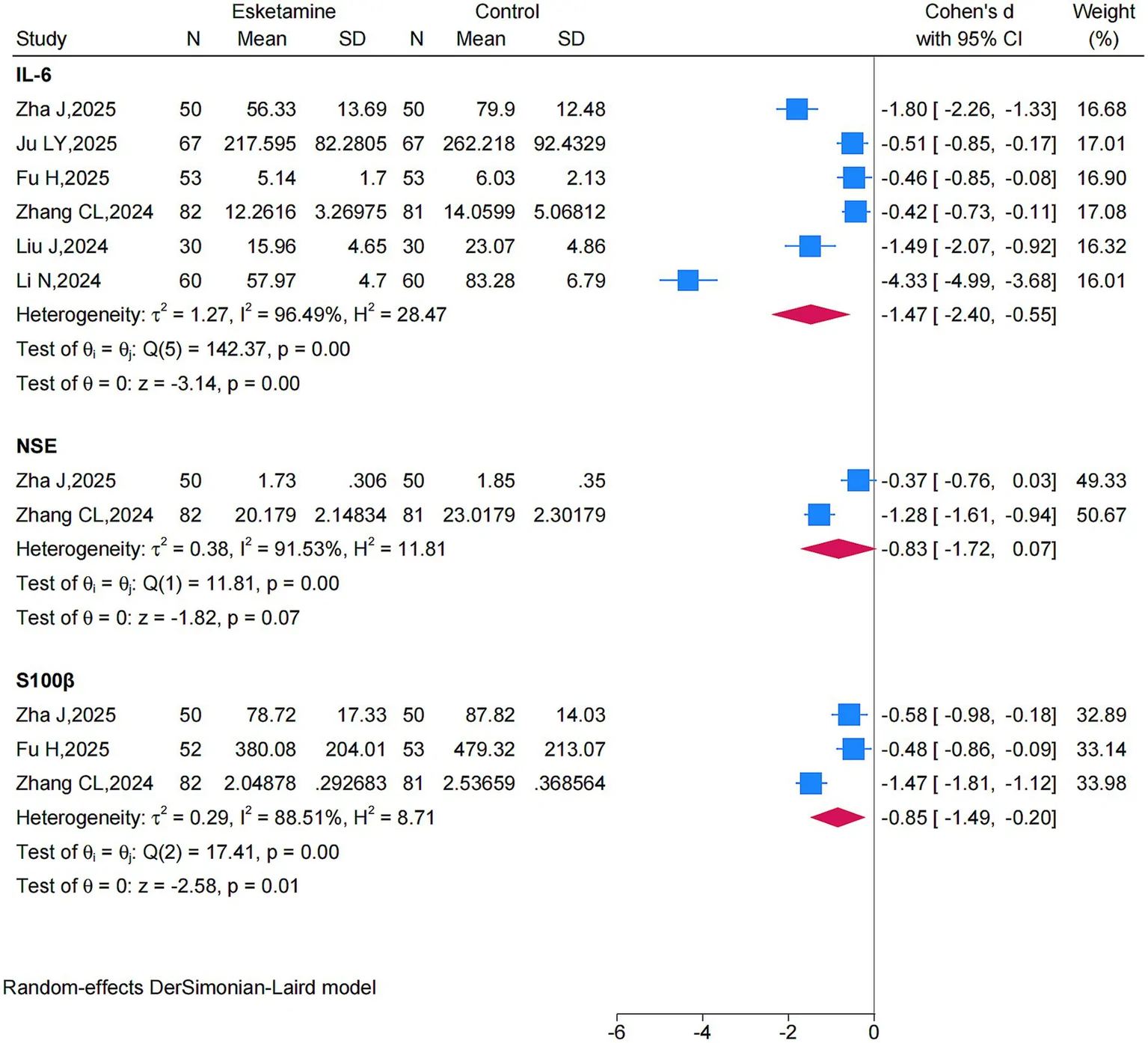
Differences in postoperative serological markers.

Inflammatory markers (e.g., IL-6) and brain injury markers (e.g., S100β and NSE) were also routinely quantified in the included studies. These biomarkers were selected for two key reasons: first, they are known to correlate with postoperative delirium (POD); second, they were intended to elucidate the potential mechanism of action of esketamine. The results indicated that esketamine administration reduced serum concentrations of IL-6 (Cohen’s d = −1.47, 95% CI: −2.40 to −0.55; *p* < 0.01) and S100β (Cohen’s d = −0.85, 95% CI: −1.49 to −0.20; *p* = 0.01) at 1 day postoperatively, whereas no statistically significant effect was observed on NSE levels (Cohen’s d = −0.83, 95% CI: −1.72 to 0.07; *p* = 0.07; Figure 8).

### Esketamine-related adverse effect

**Figure 9:**
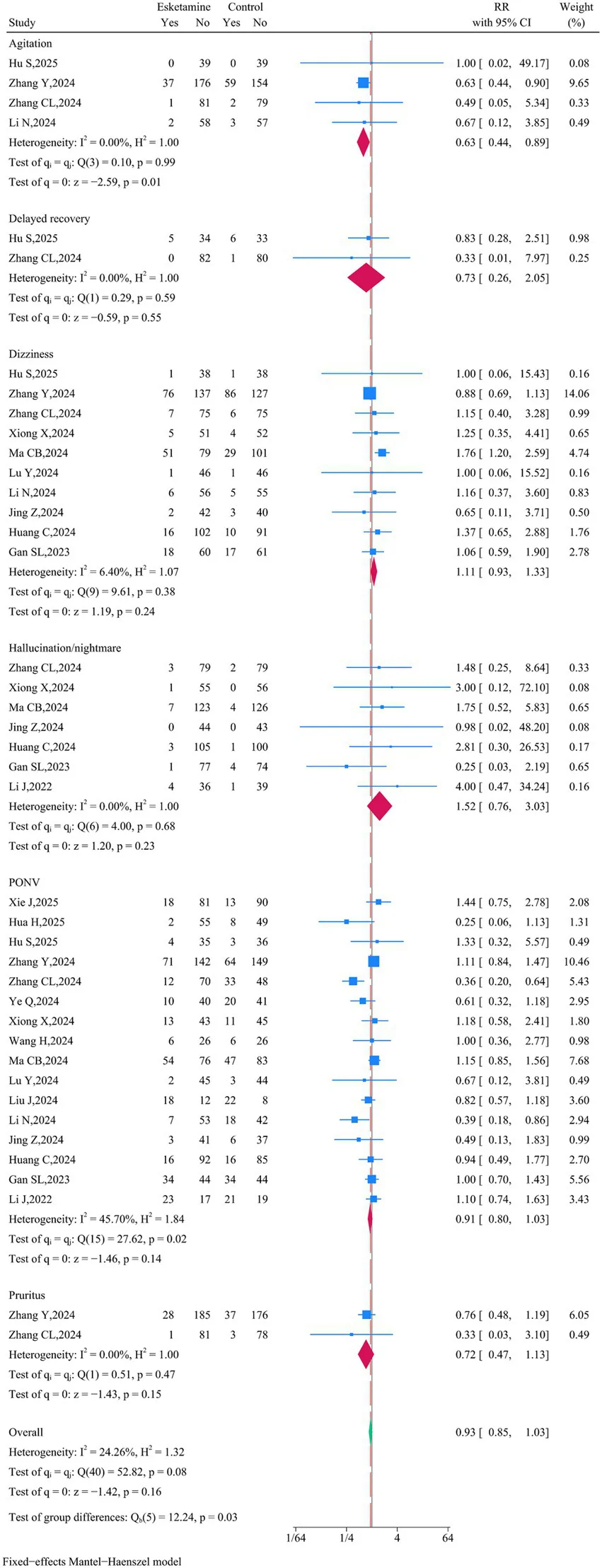
Summary of adverse events.

The adverse effects were identified in 16 articles (15–17, 24, 27, 28, 30–37, 40, 41) (Figure 9). The incidence of adverse effects was determined using dichotomous data and converted to incidence (*n*). The overall adverse effect was not significant increase in ketamine group (RR = 0.93, 95% CI, 0.85 to 1.03, *p* = 0.08). Depending on the different adverse effects associated with esketamine that may occur after surgery, we performed subgroup analyses to observe differences in nausea and vomiting, dizziness, hallucination/ nightmare, agitation, pruritus and delayed recovery between two groups. We found that esketamine could decrease the incidence of agitation (RR = 0.63, 95% CI, 0.44 to 0.89, *p* = 0.01). No statistically significant disparities were detected across all secondary adverse endpoints, with postoperative nausea and vomiting being the most commonly measured adverse event.

### Sensitivity analyses and publication bias

Funnel plot analysis of the 22 eligible RCTs showed roughly symmetric effect sizes around the pooled estimate, with no obvious skewness (Supplementary Figure 10). We quantified publication bias via two complementary methods under the DerSimonian–Laird random-effects model. Egger’s regression test yielded a coefficient of −0.73 (95% CI:−1.665 to 0.196, *p* = 0.115), showing no significant small-study effects. Trim-and-fill analysis imputed one hypothetical missing small trial on the funnel plot’s right side; after adjustment, the pooled logRR slightly attenuated from −0.562 to −0.544 with overlapping 95% CIs, indicating publication bias exerted minimal impact on our main results (Supplementary Figure 11). A leave-one-out sensitivity analysis was further performed, iteratively omitting each trial to examine its influence on the overall pooled RR for postoperative delirium. Effect magnitude and statistical significance remained stable throughout all iterations (Supplementary Figure 12). This implied that the observed protective association between esketamine and reduced postoperative delirium risk was relatively robust and not driven by any single included study.

### Certainty of evidence

The certainty of evidence for all outcomes was evaluated using the GRADE system (Table 2). The evidence supporting esketamine’s efficacy in reducing POD and dNCR incidence was judged low, mainly due to high inter-trial inconsistency and limited generalizability as all trials enrolled only Chinese patients. Evidence for MMSE and QoR-15 scores was further downgraded to very low certainty, additionally hampered by small sample sizes and wide confidence intervals. The safety outcome (adverse events) yielded low-certainty evidence, attributable to inconsistent adverse event reporting standards across trials. Collectively, only low or very low certainty evidence was available for all measured endpoints.

**Table 2:** GRADE certainty assessment of evidence for esketamine versus placebo/control drug for postoperative delirium and secondary outcomes.

Outcome	Certainty assessment							No. of patients		Effect		Certainty
	No. of studies	Study design	Risk of bias	Inconsistency	Indirectness	Imprecision	Other considerations	Esketamine	Placebo/control drug	Relative (95% CI)	Absolute (95% CI)	
POD incidence	22	Randomized trials	Not serious	Serious^a^	Serious^b^	Not serious	None	243/1432 (17.0%)	337/1428 (23.6%)	RR 0.57 (0.43 to 0.75)	101 fewer per 1,000	⨁⨁◯◯ Low^a,b^
dNCR incidence	3	Randomized trials	Not serious	Not serious	Serious^b^	Serious^c^	None	45/189 (23.8%)	72/182 (39.6%)	RR 0.60 (0.44 to 0.82)	158 fewer per 1,000	⨁⨁◯◯ Low^b,c^
MMSE score	4	Randomized trials	Not serious	Serious^a^	Serious^b^	Serious^c^	None	148	147	-	mean 0.79 higher (−0.16 to 1.74)	⨁◯◯◯ Very low^a,b,c^
QoR-15 score	4	Randomized trials	Not serious	Serious^a^	Serious^b^	Serious^c^	None	312	303	-	MD 4.8 higher (1.06 to 8.55)	⨁◯◯◯ Very low^a,b,c^
Adverse events	16	Randomized trials	Serious^d^	Not serious	Serious^b^	Not serious	None			RR 0.93 (0.85 to 1.03)	1 fewer per 1,000	⨁⨁◯◯ Low^b,d^

POD, postoperative delirium; dNCR, Delayed neurocognitive recovery; MMSE, Mini-Mental State Examination; QoR-15, 15-item Quality of Recovery questionnaire; CI, confidence interval; MD, mean difference; RR, risk ratio.

^a^There was notable inconsistency in effect estimates between different eligible RCTs.

^b^All trials originated from China, limiting the generalizability of our results to international clinical settings.

^c^The number of included studies was relatively limited.

^d^Multiple studies lacked consistent definitions and assessment standards for adverse events.

## Discussion

This meta-analysis of 22 studies involving 2,860 patients systematically evaluated the efficacy and safety of esketamine in perioperative neurocognitive protection and recovery. The key findings demonstrated that esketamine administration significantly reduced the incidence of POD by 43% compared with control groups, with this protective effect remaining consistent across diverse subgroups (including different age groups, surgical types, and dosing regimens). Additionally, esketamine exerted beneficial effects on multiple perioperative outcomes, including reducing dNCR incidence, improving short-term QoR-15 scores, lowering serum levels of inflammatory (IL-6) and brain injury (S100β) markers at 1 day postoperatively, and decreasing agitation incidence— with no clear evidence of an increased risk of overall adverse events. Taken together, these results imply a potential neuroprotective role of perioperative esketamine, with beneficial effects across multiple postoperative outcomes.

The primary finding of our study—esketamine’s significant reduction in POD incidence (RR = 0.57, 95% CI: 0.43 to 0.75)—aligns with the growing body of evidence suggesting ketamine derivatives’ neuroprotective properties. A prior meta-analysis (42) of 17 studies similarly reported a reduced POD risk following esketamine administration. Beyond updating the evidence by incorporating the latest available studies up to 2025 and validating the stability of effects across expanded subgroups, our study further strengthened the credibility of current conclusions through comprehensive sensitivity and stratified subgroup analyses to identify potential sources of heterogeneity. Additionally, we performed the GRADE certainty assessment to systematically evaluate and grade the overall quality of the synthesized evidence. For instance, Xiong et al. [15]demonstrated that a single preoperative dose of esketamine administered to patients undergoing heart valve replacement reduced the duration of delirium and the need for pharmacologic intervention for delirium symptoms. Interestingly, a study (17) involving hip surgery patients who received a higher perioperative dose of esketamine showed no significant differences in the time to delirium onset, duration of delirium, or delirium subtypes between the esketamine group and the control group; instead, it was associated with an increased incidence of postoperative dizziness. Is the occurrence of different effects attributed to dosage variations? This remains undetermined based on current clinical trials. However, these trials reveal that researchers generally tend to use low doses or subanesthetic doses in their studies. We believe this issue still requires large-scale, multicenter randomized controlled trials in real-world settings to provide answers and guide future clinical practice.

We also analyzed other indicators of interest in these articles, such as dNCR, MMSE scores, and QoR-15 scores (Figures 6, 7). Notably, these findings are consistent with those of recently published meta-analyses focusing on dNCR (43) and quality of recovery after surgery (44). Sun et al. (43) found that perioperative esketamine was associated with a reduced risk of dNCR (RR: 0.41, 95% CI: 0.21 to 0.78, *p* < 0.001) but no statistically significant difference in the risk of PND at 3 months post-surgery. Similarly, perioperative use of ketamine/esketamine is associated with improved early subjective QoR (44, 45). Although both improvements in short-term cognitive function and quality of recovery have been observed, there is a growing focus on patients’ long-term prognosis, with an increasing number of studies incorporating 1-month, 3-month, or even longer follow-up data. Notably, several studies within our inclusion criteria additionally investigated esketamine’s impacts on perioperative sleep (17, 31, 33, 36), anxiety/depression (14–16, 32, 33, 37, 40, 41), and the majority reported positive clinical outcomes. That said, the extensive diversity of assessment instruments for these psychological and sleep-related endpoints, combined with conflicting scoring conventions (i.e., higher scores may denote either improved or impaired status depending on the scale), posed insurmountable barriers to standardized quantitative synthesis. Even so, existing trails (46–48) collectively highlight esketamine’s promising potential for enhancing postoperative sleep quality and mitigating anxiety and symptoms in surgical patients.

We further subdivided the included studies into refined surgical subgroups to explore potential effect modification (Figure 5; Supplementary Figure 9). Subgroup analyses revealed that the magnitude of the protective effect of esketamine against postoperative delirium varied markedly across these finely categorized surgical types, indicating inconsistent treatment effects among subgroups. Specifically, significant reductions in postoperative delirium risk were observed in cardiac, orthopedic, and abdominal surgical subgroups. However, the relatively small sample size within each surgical subgroup poses challenges to the robustness of these subgroup findings. Such discrepancies may arise from inherent distinctions across surgical procedures, including divergent inflammatory burden, ICU exposure duration, postoperative pain intensity, and cumulative perioperative opioid consumption (49).

A robust body of evidence has confirmed that perioperative neurocognitive disorder is tightly linked to neuroinflammation and neuronal damage (3, 50). IL-6 emerged as the most commonly utilized inflammatory biomarker in our meta-analysis, with six constituent trials evaluating postoperative circulating IL-6 concentrations (Figure 8). Prior studies have confirmed that a single low dose of esketamine delivered at induction effectively alleviates early postoperative depression and anxiety, with the drug’s anti-inflammatory potential (encompassing neuroinflammation modulation and neurotrophic signaling promotion) proposed as the core mediating pathway for this effect (51). Our meta-analysis further corroborated its modulatory effects on perioperative inflammation: all included trials demonstrated that esketamine treatment led to a statistically significant decrease in postoperative IL-6 levels (Cohen’s d = −1.47, 95% CI: −2.40 to −0.55; *p* < 0.01). In addition to IL-6, a subset of studies extended assessments to other proinflammatory mediators (CPR (26), TNF-*α* (35), IL-1β (36), IL-8 (35)) and the anti-inflammatory cytokine IL-10 (30), providing a broader profile of esketamine’s immunomodulatory activity. Nevertheless, the limited specificity of these inflammatory markers and their strong susceptibility to perturbation by concurrent inflammatory diseases and brain dysfunction preclude their utility as standalone surrogate indicators for POD/PND. Neuronal injury is hypothesized to represent a “common pathway” underlying the multifactorial pathogenesis of POD (52), while serum S100β and NSE are widely used biomarkers for reflecting perioperative neuronal damage. In the present meta-analysis, esketamine administration was associated with a statistically significant reduction in postoperative day 1 serum S100β levels (Cohen’s d = −0.85, 95% confidence interval [CI]: −1.49 to −0.20; *p* = 0.01); by contrast, no significant effect on perioperative NSE concentrations was observed (Cohen’s d = −0.83, 95% CI: −1.72 to 0.07; *p* = 0.07).

A particularly striking and unanticipated observation from this meta-analysis was that esketamine significantly lowered the incidence of postoperative agitation (RR = 0.63, 95% CI: 0.44 to 0.89, *p* = 0.01). Numerous clinical RCTs have corroborated that perioperative esketamine use confers a protective effect against the development of emergence agitation in the pediatric postoperative population (53–55).

No statistically significant disparities were detected across all secondary adverse endpoints, with postoperative nausea and vomiting being the most commonly measured adverse event. Notably, several trials lacked clear standardized definitions, uniform monitoring windows, and complete tabulation of event counts for individual adverse reactions, which restricts definitive conclusions regarding comparative safety profiles between groups.

This meta-analysis has several limitations that should be acknowledged. First, although the present study included 22 latest available trials with a larger overall sample size than previous relevant meta-analyses, all eligible studies were exclusively conducted in Chinese populations. Variations in patient baseline characteristics, surgical protocols, and baseline perioperative delirium risk across different regions may affect the efficacy of esketamine, thereby limiting the generalizability of our findings to global clinical settings. Second, substantial heterogeneity was observed across the included studies, which could be partially attributed to variable surgical types, esketamine administration regimens, patient age distributions, and inconsistent assessment criteria for postoperative delirium and adverse events. Although subgroup analyses revealed that follow-up duration and surgical type were major sources of heterogeneity, these factors could not fully account for the between-study variability. Third, due to the considerable diversity in esketamine dosage and administration modes across trials, we were unable to perform a dose–response meta-analysis to clarify the quantitative association between esketamine exposure and clinical outcomes. Fourth, regional single-population restriction, inherent clinical heterogeneity, and the limited number of studies for several secondary outcomes compromised the overall certainty of the synthesized evidence. Correspondingly, the GRADE assessment rated most outcome indicators as low or very low certainty evidence, which weakens the reliability and extrapolation of our conclusions.

The following perspectives are proposed for future research investigating the effects of esketamine on POD. First, to more accurately and comprehensively characterize patients’ perioperative neurocognitive status, standardized and unified assessment criteria for the classification, severity, and duration of postoperative delirium should be established. Clinically, POD assessments should be performed for a minimum of 3 postoperative days, ideally for 7 consecutive days, with no fewer than two independent evaluations conducted daily. Second, given the substantial heterogeneity in current esketamine administration regimens, dedicated high-quality studies are urgently required to determine the optimal dosage and administration timing tailored to distinct surgical populations and procedures. In particular, dose–response relationship analyses are essential to identify the clinically optimal dose and treatment duration for standardized clinical application. Third, existing evidence regarding the long-term neurocognitive outcomes following esketamine administration remains insufficient. Future clinical trials should prioritize long-term follow-up observations to clarify the sustained impact of esketamine on postoperative cognitive function. Finally, well-designed international multicenter randomized controlled trials are urgently needed to address the geographic homogeneity of current evidence and further validate the efficacy of esketamine against POD across diverse populations, thereby establishing standardized guidelines for its generalized clinical application in postoperative delirium management.

## Conclusion

In conclusion, this meta-analysis suggests that perioperative esketamine administration is associated with reduced incidence of POD and dNCR, improved short-term postoperative recovery quality, alleviated neuroinflammation and neuronal injury, and decreased postoperative agitation, with no obvious elevation in overall adverse event risk. These beneficial trends observed across different patient subgroups and surgical categories indicate the promising potential of esketamine as a multifunctional neuroprotective agent for perioperative application. Nevertheless, current evidence is limited by clinical heterogeneity, geographical restriction, and insufficient standardized assessment protocols. Future high-quality studies with unified dosing regimens, rigorous and comprehensive neurocognitive evaluation systems, and prolonged follow-up periods are warranted to further validate and refine the clinical value of esketamine in optimizing perioperative patient care.

## Data Availability

The original contributions presented in the study are included in the article/Supplementary material, further inquiries can be directed to the corresponding author.

## Supplementary material

SUPPLEMENTARY FIGURE 1 Forest plot of the incidence of POD between esketamine and control group (REML random-effects model).

SUPPLEMENTARY FIGURE 2 Forest plot of the incidence of POD between esketamine and control group (Sidik-Jonkman random-effects model).

SUPPLEMENTARY FIGURE 3 Forest plot of the incidence of POD following removal of double-zero event trials.

SUPPLEMENTARY FIGURE 4 Forest plot of the incidence of POD following removal of combination-treatment trials.

SUPPLEMENTARY FIGURE 5 Subgroup analysis by assessment duration.

SUPPLEMENTARY FIGURE 6 Subgroup analysis by age.

SUPPLEMENTARY FIGURE 7 Subgroup analysis by esketamine administration regimen.

SUPPLEMENTARY FIGURE 8 Subgroup analysis by endpoint priority.

SUPPLEMENTARY FIGURE 9 Subgroup analysis by surgical type.

SUPPLEMENTARY FIGURE 10 Funnel plot.

SUPPLEMENTARY FIGURE 11 Trim-and-fill funnel plot.

SUPPLEMENTARY FIGURE 12 Sensitivity analysis.

## Glossary

POD: Postoperative delirium
RCTs: Randomized controlled trials
CI: Confidence intervals
RR: Risk ratios
MD: Mean differences
Cohen’s d: Standardized mean differences
dNCR: Delayed neurocognitive recovery
POCD: Postoperative cognitive dysfunction
PND: Postoperative neurocognitive disorder
NMDA: N-methyl-D-aspartate
QoR: Quality of Recovery
NSE: Neuron-specific enolase
S100*β*: Central nervous system specific protein β
ASA: American Society of Anesthesiologists
PCIA: Patient-controlled intravenous analgesia
TKA: Total knee arthroplasty
THA: Total hip arthroplasty
OPCABG: Off-pump coronary artery bypass grafting
ICDSC: Intensive Care Delirium Screening Checklist
CAM-CR: Chinese Revised Delirium Diagnostic Scale
3D-CAM: 3-min Diagnostic Confusion Assessment Method
CAM: Confusion Assessment Method
CAM-ICU: Confusion Assessment Method for the Intensive Care Unit
MMSE: Mini-Mental State Examination
GRADE: Grading of Recommendations Assessment, Development and Evaluation
REML: Restricted Maximum Likelihood

## References

[1] Jin Z Hu J Ma D. Postoperative delirium: perioperative assessment, risk reduction, and management. Br J Anaesth. (2020) 125:492–504. doi: 10.1016/j.bja.2020.06.063, 32798069

[2] Li Y Li Z Lv Q Gu Y Qi Y Li J . Prevalence and risk factors of postoperative delirium in tumor patients after free flap reconstruction: a systematic review and meta-analysis of case-control studies. Surgery. (2024) 176:906–17. doi: 10.1016/j.surg.2024.05.009, 38910046

[3] Liu J Li C Yao J Zhang L Zhao X Lv X . Clinical biomarkers of perioperative neurocognitive disorder: initiation and recommendation. Sci China Life Sci. (2025) 68:1912–40. doi: 10.1007/s11427-024-2797-x 39918707

[4] Yan E Veitch M Saripella A Alhamdah Y Butris N Tang-Wai DF . Association between postoperative delirium and adverse outcomes in older surgical patients: a systematic review and meta-analysis. J Clin Anesth. (2023) 90:111221. doi: 10.1016/j.jclinane.2023.111221 37515876

[5] Lander HL Dick AW Joynt Maddox KE Oldham MA Fleisher LA Mazzeffi M . Postoperative delirium in older adults undergoing noncardiac surgery. JAMA Netw Open. (2025) 8:e2519467. doi: 10.1001/jamanetworkopen.2025.19467, 40627352 PMC12238904

[6] Fong TG Inouye SK. The inter-relationship between delirium and dementia: the importance of delirium prevention. Nat Rev Neurol. (2022) 18:579–96. doi: 10.1038/s41582-022-00698-7, 36028563 PMC9415264

[7] Fong TG Davis D Growdon ME Albuquerque A Inouye SK. The interface between delirium and dementia in elderly adults. Lancet Neurol. (2015) 14:823–32. doi: 10.1016/S1474-4422(15)00101-5, 26139023 PMC4535349

[8] Evered LA Chan MTV Han R Chu MHM Cheng BP Scott DA . Anaesthetic depth and delirium after major surgery: a randomised clinical trial. Br J Anaesth. (2021) 127:704–12. doi: 10.1016/j.bja.2021.07.021, 34465469 PMC8579421

[9] Wei Y Chang L Hashimoto K. Molecular mechanisms underlying the antidepressant actions of arketamine: beyond the NMDA receptor. Mol Psychiatry. (2022) 27:559–73. doi: 10.1038/s41380-021-01121-1, 33963284 PMC8960399

[10] Ren L Zhang T Zou B Su X Tao Y Yang J . Intraoperative Esketamine and postpartum depression among women with cesarean delivery: a randomized clinical trial. JAMA Netw Open. (2025) 8:e2459331. doi: 10.1001/jamanetworkopen.2024.59331, 39946130 PMC11826358

[11] Fountoulakis KN Saitis A Schatzberg AF. Esketamine treatment for depression in adults: a PRISMA systematic review and meta-analysis. Am J Psychiatry. (2025) 182:259–75. doi: 10.1176/appi.ajp.20240515, 39876682

[12] Qiu D Wang XM Yang JJ Chen S Yue CB Hashimoto K . Effect of intraoperative esketamine infusion on postoperative sleep disturbance after gynecological laparoscopy: a randomized clinical trial. JAMA Netw Open. (2022) 5:e2244514. doi: 10.1001/jamanetworkopen.2022.44514, 36454569 PMC9716381

[13] Wang S Deng CM Zeng Y Chen XZ Li AY Feng SW . Efficacy of a single low dose of esketamine after childbirth for mothers with symptoms of prenatal depression: randomised clinical trial. BMJ. (2024) 385:e078218. doi: 10.1136/bmj-2023-078218 38808490 PMC11957566

[14] Zha J Chen H Sun Z Shi R Yan R Guo L . Effects of Esketamine on postoperative delirium and postoperative cognitive function in elderly gastrointestinal tumor patients with preoperative anxiety. Drug Des Devel Ther. (2025) 19:9425–37. doi: 10.2147/DDDT.S528902, 41127368 PMC12539357

[15] Xiong X Shao Y Chen D Chen B Lan X Shi J. Effect of esketamine on postoperative delirium in patients undergoing cardiac valve replacement with cardiopulmonary bypass: a randomized controlled trial. Anesth Analg. (2024) 139:743–53. doi: 10.1213/ANE.0000000000006925, 38446699

[16] Zhang Y Chen R Tang S Sun T Yu Y Shi R . Diurnal variation of postoperative delirium in elderly patients undergoing esketamine anesthesia for elective noncardiac surgery: a randomized clinical trial. Int J Surg. (2024) 110:5496–504. doi: 10.1097/JS9.0000000000001642, 39275772 PMC11392167

[17] Ma CB Zhang CY Gou CL Liang ZH Zhang JX Xing F . Effect of low-dose Esketamine on postoperative delirium in elderly patients undergoing Total hip or knee arthroplasty: a randomized controlled trial. Drug Des Devel Ther. (2024) 18:5409–21. doi: 10.2147/DDDT.S477342, 39624771 PMC11609288

[18] Brenna CTA He XW Liu D Kaustov L Choi S Orser BA. Effects of ketamine on postoperative cognition: a scoping review. Br J Anaesth. (2025) 135:642–59. doi: 10.1016/j.bja.2025.05.035, 40628582 PMC12489380

[19] Page MJ McKenzie JE Bossuyt PM Boutron I Hoffmann TC Mulrow CD . The PRISMA 2020 statement: an updated guideline for reporting systematic reviews. BMJ. (2021) 372:n71. doi: 10.1136/bmj.n71 33782057 PMC8005924

[20] Sterne JAC Savović J Page MJ Elbers RG Blencowe NS Boutron I . RoB 2: a revised tool for assessing risk of bias in randomised trials. BMJ. (2019) 366:l4898. doi: 10.1136/bmj.l4898, 31462531

[21] Guyatt G Oxman AD Akl EA Kunz R Vist G Brozek J . GRADE guidelines: 1. Introduction-GRADE evidence profiles and summary of findings tables. J Clin Epidemiol. (2011) 64:383–94. doi: 10.1016/j.jclinepi.2010.04.026, 21195583

[22] Guyatt GH Oxman AD Vist GE Kunz R Falck-Ytter Y Alonso-Coello P . GRADE: an emerging consensus on rating quality of evidence and strength of recommendations. BMJ. (2008) 336:924–6. doi: 10.1136/bmj.39489.470347.AD, 18436948 PMC2335261

[23] Guyatt GH Oxman AD Schünemann HJ Tugwell P Knottnerus A. GRADE guidelines: a new series of articles in the journal of clinical epidemiology. J Clin Epidemiol. (2011) 64:380–2. doi: 10.1016/j.jclinepi.2010.09.011, 21185693

[24] Xie J Shen F Wang X Yao J Zhou L Huang L . The effect of minimal-dose S-ketamine administration post-surgery on opioids consumption and functional rehabilitation exercises in patients undergoing minimally invasive radical resection of esophageal cancer. Ther Clin Risk Manag. (2025) 21:1033–44. doi: 10.2147/TCRM.S527262, 40641594 PMC12241663

[25] Xi L Liu H Tang X Jiang Z. Effects of esketamine combined with remimazolam tosylate on hemodynamics during cardiovascular Anesthesia. Clin Transl Sci. (2025) 18:e70232. doi: 10.1111/cts.70232, 40720250 PMC12302974

[26] Ju LY Liu GQ Yuan L Lyu L Su Y Liu AJ. Effect of esketamine on postoperative delirium and inflammatory response in patients undergoing off-pump coronary artery bypass grafting: a randomized controlled study. J Cardiothorac Vasc Anesth. (2025) 39:2079–87. doi: 10.1053/j.jvca.2025.04.013 40320348

[27] Hua H He T Li X Chen X Liu Z Liu K . Effects of esketamine-mediated opioid-free anesthesia on delirium in elderly patients after hip replacement. Chinese J Clin Pharmacol Therap. (2025) 30:78–84. doi: 10.12092/j.issn.1009-2501.2025.01.009

[28] Hu S Cheng J Fang Y Cao G Jiang T Wang Y. Effect of low-dose esketamine on intraoperative electroencephalographic burst suppression under general anesthesia in elderly patients. Chinese J Anesthesiol. (2025) 45:703–8. doi: 10.3760/cma.j.cn131073-20240816-00607

[29] Fu H Hu J Zhang X Xie K Hu L. The effects of esketamine on postoperative delirium in older patients with fragile brain function during the non-acute phase following lung cancer surgery: a randomized controlled trial. BMC Geriatr. (2025) 25:608. doi: 10.1186/s12877-025-06268-y, 40783518 PMC12335161

[30] Zhang CL Yan Y Zhang Y Bai HL Zhuang Q Song NN . Effects of esketamine combined with dexmedetomidine on postoperative delirium and quality of recovery in elderly patients undergoing thoracoscopic radical lung cancer surgery: a randomized controlled trial. CNS Spectr. (2024) 29:441–450. doi: 10.1017/S1092852924002177 39564615

[31] Ye Q Hu Y Xing Q Wu Y Zhang Y. The effects of opioid-free Anesthesia with dexmedetomidine and Esketamine on postoperative Anesthetic-related complications for hip surgery in the elderly. Int J General Med. (2024) 17:6291–302. doi: 10.2147/IJGM.S492771, 39712198 PMC11662907

[32] Wang H Te R Zhang J Su Y Zhou H Guo N . Effects of a single subanesthetic dose of esketamine on postoperative subthreshold depressive symptoms in patients undergoing unilateral modified radical mastectomy: a randomised, controlled, double-blind trial. BMC Psychiatry. (2024) 24:315. doi: 10.1186/s12888-024-05753-9, 38658886 PMC11044398

[33] Lu Y Yin G Jin C Gu K Bao D Xu W . The application value of esketamine and dexmedetomidine in preventing postoperative delirium and hyperalgesia in elderly patients with thoracic anesthesia. Altern Ther Health Med. (2024) 30:80–5. 37917895

[34] Liu J Wang T Song J Cao L. Effect of esketamine on postoperative analgesia and postoperative delirium in elderly patients undergoing gastrointestinal surgery. BMC Anesthesiol. (2024) 24:46. doi: 10.1186/s12871-024-02424-w, 38302882 PMC10832082

[35] Li N Zhang H Zhou J. Effect of sub-anesthetic dose of esketamine on chronic post-surgery pain in patients undergoing radical mastectomy of breast cancer. Chin J Clin Pharmacol Ther. (2024) 29:671–9. doi: 10.12092/j.issn.1009-2501.2024.06.009

[36] Jing Z Han Y Li Y Zeng R Wu J Wang Y . Effect of subanesthetic dose of esketamine on postoperative pain in elderly patients undergoing laparoscopic gastrointestinal tumor surgery: a prospective, double-blind, randomized controlled trial. Heliyon. (2024) 10:e27593. doi: 10.1016/j.heliyon.2024.e27593, 38495154 PMC10943442

[37] Huang C Yang R Xie X Dai H Pan L. Effect of small dose esketamine on perioperative neurocognitive disorder and postoperative depressive symptoms in elderly patients undergoing major elective noncardiac surgery for malignant tumors: a randomized clinical trial. Medicine. (2024) 103:e40028. doi: 10.1097/MD.0000000000040028, 39432604 PMC11495772

[38] Ma J Wang F Wang J Wang P Dou X Yao S . The effect of low-dose Esketamine on postoperative neurocognitive dysfunction in elderly patients undergoing general anesthesia for gastrointestinal tumors: a randomized controlled trial. Drug Des Devel Ther. (2023) 17:1945–57. doi: 10.2147/DDDT.S406568, 37408867 PMC10318106

[39] Liu T Zhang X Li A Liu T Yang X Zhang H . Effects of intra-operative administration of subanesthetic s-ketamine on emergence from sevoflurane anesthesia: a randomized double-blind placebo-controlled study. BMC Anesthesiol. (2023) 23:221. doi: 10.1186/s12871-023-02170-5, 37353750 PMC10288804

[40] Gan SL Long YQ Wang QY Feng CD Lai CX Liu CT . Effect of esketamine on postoperative depressive symptoms in patients undergoing thoracoscopic lung cancer surgery: a randomized controlled trial. Front Psych. (2023) 14:14. doi: 10.3389/fpsyt.2023.1128406, 37009103 PMC10050377

[41] Li J Wang Z Wang A Wang Z. Clinical effects of low-dose esketamine for anaesthesia induction in the elderly: a randomized controlled trial. J Clin Pharm Ther. (2022) 47:759–66. doi: 10.1111/jcpt.13604, 35018643

[42] Zhang W Wang D Li S Chen Y Bi C. Effect of esketamine on postoperative delirium in general anesthesia patients undergoing elective surgery: a meta-analysis of randomized controlled trials. BMC Anesthesiol. (2024) 24:442. doi: 10.1186/s12871-024-02833-x, 39609668 PMC11603621

[43] Su X Chen L Zhao Y Li C Liu S Wang Z . Impact of perioperative esketamine on the perioperative neurocognitive dysfunction: a systematic review and meta-analysis of randomised controlled studies. BMJ Open. (2025) 15:e095695. doi: 10.1136/bmjopen-2024-095695, 40398929 PMC12096981

[44] Hung KC Kao CL Ho CN Hsing CH Chang YJ Wang LK . The impact of perioperative ketamine or esketamine on the subjective quality of recovery after surgery: a meta-analysis of randomised controlled trials. Br J Anaesth. (2024) 132:1293–303. doi: 10.1016/j.bja.2024.03.012, 38614917

[45] Zhang J Wang F Dang J Zheng H Ren B Liu C . Effect of intraoperative infusion of Esketamine on quality of postoperative recovery in patients undergoing laparoscopic bariatric surgery: a randomized controlled trial. Pain Ther. (2023) 12:979–92. doi: 10.1007/s40122-023-00519-9, 37171754 PMC10289955

[46] Song Z Miao M Huang F Hu L Yang X Cui W . Effect of a single low-dose esketamine administration during surgical abortion on postoperative sleep disturbance: a randomized controlled trial. Nat Commun. (2025) 16:7533. doi: 10.1038/s41467-025-62933-1, 40804260 PMC12350747

[47] Zhou YH Li L Gao R Yang YJ Yan S Wang Q . Effect of low-dose supplemental esketamine infusion on the intraoperative frontal electroencephalography dynamics and postoperative sleep disturbance during gynecological laparoscopic surgery: a double-blind, randomized clinical trial. Int J Surg. (2025) 111:6988–7000. doi: 10.1097/JS9.0000000000002836, 40548668 PMC12527740

[48] Shen M Xuan Y Chen K Lei W Huang Y Wang J . Effects of continuous perioperative Esketamine infusion on postoperative depression in breast cancer patients: a randomized controlled double-blind trial. Drug Des Devel Ther. (2025) 19:9685–95. doi: 10.2147/DDDT.S540781, 41185705 PMC12579836

[49] Aldecoa C Bettelli G Bilotta F Sanders RD Aceto P Audisio R . Update of the European Society of Anaesthesiology and Intensive Care Medicine evidence-based and consensus-based guideline on postoperative delirium in adult patients. Eur J Anaesthesiol. (2024) 41:81–108. doi: 10.1097/EJA.0000000000001876, 37599617 PMC10763721

[50] Leng K Maze M Barreto Chang OL. Emerging biomarkers of postoperative delirium at the intersection of neuroinflammation and neurodegeneration. Front Aging Neurosci. (2025) 17:1632947. doi: 10.3389/fnagi.2025.1632947, 40951922 PMC12426202

[51] Zhang ZN Hao XY Cai C Sun L Zhang ZY Wang M . Effect of esketamine on postoperative depression and anxiety in patients undergoing cardiac valve surgery: a randomised, placebo-controlled, double-blinded clinical trial. Pharmacol Res. (2025) 222:108047. doi: 10.1016/j.phrs.2025.108047, 41285329

[52] Taylor J Parker M Casey CP Tanabe S Kunkel D Rivera C . Postoperative delirium and changes in the blood-brain barrier, neuroinflammation, and cerebrospinal fluid lactate: a prospective cohort study. Br J Anaesth. (2022) 129:219–30. doi: 10.1016/j.bja.2022.01.005, 35144802 PMC9465948

[53] Lin H Chen S Shi X Yang J Gao W Yao Y. Intravenous esketamine for the prevention of emergence delirium and negative behavioural changes after paediatric adenotonsillectomy: a randomised controlled trial. Anaesthesia. (2026) 81:232–9. doi: 10.1111/anae.70018, 41039865

[54] Li J Jia G Wang R Zheng R Yuan K. The impact of intranasal Esketamine on emergence agitation in children undergoing adenotonsillectomy: a randomized controlled study. Drug Des Devel Ther. (2025) 19:10109–17. doi: 10.2147/DDDT.S553693, 41256828 PMC12621606

[55] Jiang P Liu W Peng Q Feng Y Wang D Luo K . Compare the effects of 0 mg/kg, 0.1 mg/kg, 0.3 mg/kg and 0.5 mg/kg Esketamine on emergence agitation after tonsillectomy and adenoidectomy in children: a randomized control trial. Drug Des Devel Ther. (2025) 19:6543–52. doi: 10.2147/DDDT.S525687, 40756268 PMC12318529

